# Reduced expression of Twist 1 is protective against insulin resistance of adipocytes and involves mitochondrial dysfunction

**DOI:** 10.1038/s41598-018-30820-z

**Published:** 2018-08-22

**Authors:** Sumei Lu, Hong Wang, Rui Ren, Xiaohong Shi, Yanmei Zhang, Wanshan Ma

**Affiliations:** 1 Department of Laboratory Medicine, Shandong Provincial Qianfoshan Hospital, Shandong University, Jinan, Shandong 250014 P. R. China; 2 Otolaryngology-Head and Neck Surgery, Shandong Provincial Qianfoshan Hospital, Shandong University, Jinan, Shandong 250014 P. R. China

## Abstract

Insulin resistance (IR) has become a global epidemic that represents a serious hazard to public health. However, the precise mechanisms modulating IR have not been fully elucidated. The present study aimed to investigate the role of transcriptional factor Twist 1 in adipocyte IR and to further explore the molecular mechanism. An *in vitro* IR model based on cultured 3T3-L1 adipocytes was established under high glucose/insulin stimulation and an *in vivo* IR model in C57/BL6J mice induced by a high fat diet (HFD) was also developed. Lentivirus targeting Twist 1 silencing was introduced. The relationships between Twist 1 expression and IR state, mitochondrial dysfunction and the downstream insulin signaling pathway were assayed. Our results firstly showed the elevation of Twist 1 in IR adipocytes, and Twist 1 silencing attenuated IR. Then mitochondrial ultra-structural damage, elevated ROS, decreased MMP and ATP, and changes in mitochondrial biosynthesis-related genes in IR group indicated mitochondrial dysfunction. Further, the downstream IRS/PI3K/AKT/GluT4 pathway was showed involved in Twist 1-mediated IR. In total, we provide evidence of a protective role of Twist 1 silencing in relieving the IR state of adipocytes. Mitochondrial dysfunction and the downstream IRS/PI3K/AKT/GluT4 pathway were involved in this Twist 1-mediated IR.

## Introduction

Insulin resistance (IR) is characterized by decreased sensitivity of insulin target tissues (adipose, muscles, and hepatocytes) to normal concentrations of insulin, resulting in reduced ability of the body to control glucose^1–3^. Studying IR in adipose has recently attracted significant attention due to the high incidence and serious impact of obesity and diabetes, and interest in this area continues to grow. It has been reported that IR increases the risks of obesity^4^, type 2 diabetes^5^, cardiovascular diseases^6^, metabolic syndrome^7^, and islet β cell early exhaustion^8^, etc. However, the precise mechanisms regarding the regulation of IR have not been fully clarified.

Twist 1, a member of the basic helix-loop-helix (bHLH) super-family, plays critical roles in regulating the development of tissues and organs due to interactions between its specific bHLH domain and target DNA sequences^9–11^. Previous studies have reported that Twist 1 can be detected in brown and white adipocytes and that its expression is correlated with the homeostasis model assessment of insulin resistance (HOMA-IR) and body mass index (BMI)^12^. Twist 1 was also shown to be involved in regulating adipocyte metabolism, which occurs mainly in mitochondria^13,14^. A recent study showed that Twist 1 interacted with PGC-1α, which was important in mitochondria synthesis and function and could inhibit UCP1 in brown adipocytes, which was involved in the mitochondrial oxidative metabolism and uncoupling process^12^. Additionally, Twist 1 knockdown was found to induce the expression of UCP1 and CPT-1, which are related to oxidative metabolism, and to increase PGC-1α expression and mediate mitochondrial biogenesis in brown adipocytes^15,16^. These findings indicate the role of Twist 1 in IR and mitochondria-mediated adipocyte metabolism.

Mitochondria are believed to be critical in the occurrence and development of adipocyte IR^17,18^. Significant mitochondrial metabolic disorders are often observed in adipose tissue of diabetic patients or individuals with a family history of diabetes^19–21^. A decrease in mitochondrial biosynthesis gene expression occurs in the pre-diabetic state^22–24^. The insulin sensitizer pioglitazone can improve the insulin sensitivity of adipocytes by promoting mitochondrial biosynthesis and regulating mitochondrial oxidative capacity^25–27^. Taken together, existing studies have strongly indicated the potential roles of Twist 1 in the pathogenesis of IR. However, the molecular mechanism involved in Twist 1 and IR has not yet been fully elucidated, especially the mitochondria-related molecular mechanisms.

Additionally, the establishment of ideal IR model is critical for research, especially for animal experiment. Several typical methods were applied in IR model establishment based on animals, during which continuous feeding of high fat diet (HFD) is the most popular, and the composition of HFD is critical for the model building. HFD produced by Research Diets (No: D12492, USA) has been considered the standard diet for obesity, diabetes and metabolic syndrome, and is the most popular diet in IR studies. The main fat composition of HFD (No: D12492) is lard (245 gm) and soybean oil (25 gm), with 60% calories, which is the limit from the nutrition. Here we selected this HFD (No: D12492) from Research Diets to establish the IR model.

In the present study, we established an *in vitro* IR model based on cultured mouse 3T3-L1 adipocytes by high glucose/insulin stimulation and an *in vivo* model in C57/BL6J mice induced by feeding HFD (No: D12492). Twist 1 lentiviral silencing was applied to determine the function of Twist 1 in insulin sensitivity. The results showed the protective effect of Twist 1 silencing against IR. We further found that the mitochondria ultrastructure, mitochondria synthesis-related genes, and mitochondrial function (ROS, MMP, ATP) were involved in IR based on Twist 1. The present study revealed the role of Twist 1 silencing in enhancing insulin sensitivity by antagonizing mitochondrial damage and suggests Twist 1 as a regulatory gene in IR of adipocytes. Strategies targeting Twist 1 silencing maybe effective for enhancing adipocyte insulin sensitivity.

## Results

### Twist 1 expression was elevated in IR cells and interference of Twist 1 attenuated the IR state of 3T3-L1 adipocytes

**Figure 1:**
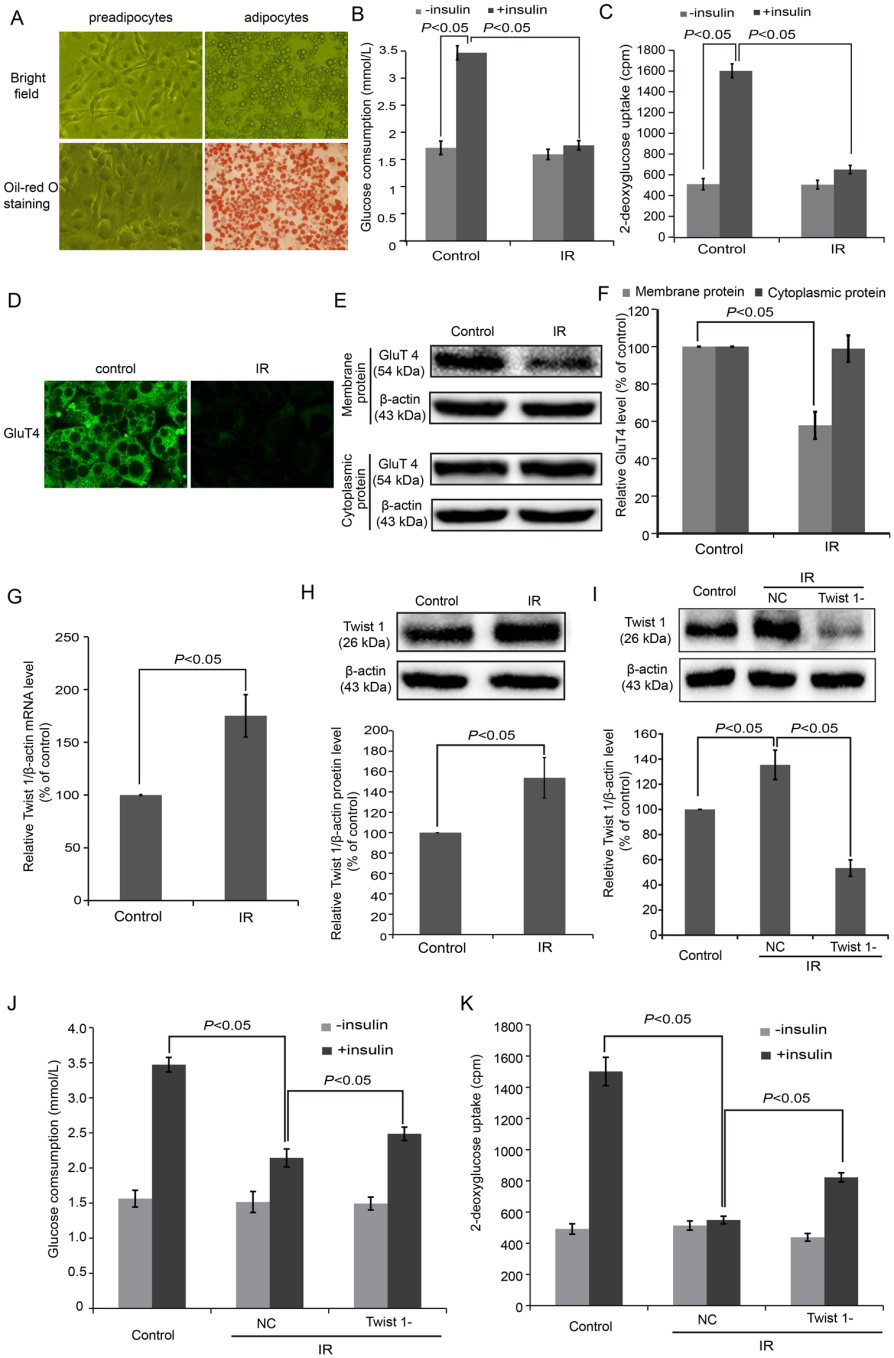
An *in vitro* IR model was successfully established in cultured 3T3-L1 adipocytes. (**A**) Morphological observations and Oil red O staining of 3T3-L1 adipocytes. (**B**) Glucose consumption test showed low levels of glucose under insulin stimulation in IR cells. (**C**) Glucose uptake assay showed low levels of glucose uptake under insulin stimulation in IR cells. (**D**) Immunofluorescence detection revealed inhibition of GluT4 membrane translocation in IR cells. (**E**) GluT4 expression in the cell membranes decreased in IR cells according to western blot. (**F**) The semi-quantification of GluT4 (**E**) by western blot and using ImageJ software. (**G**) The mRNA of Twist 1 was determined in IR cells based on real-time PCR. (**H**) The expression of Twist 1 was determined in IR cells based on western blot. (**I**) The siRNA induced an obvious Twist 1 silencing effect according to western blot. (**J**) Glucose uptake tests revealed elevated glucose in Twist 1-silenced cells. (**K**) Glucose consumption assay also showed elevated glucose in Twist 1-silenced cells.

To investigate the role of Twist 1 in IR, we first established an IR model based on 3T3-L1 adipocytes *in vitro* by high glucose/insulin stimulation. Obvious lipid droplets could be observed in the 3T3-L1 adipocytes on days 10–12 of differentiation, and more than 95% of adipocytes were stained by Oil red O (Fig. 1A). A glucose consumption test (Fig. 1B) was conducted between control and IR cells. In control cells, the glucose consumption differed by nearly 2.01-fold between cells with and without insulin stimulation (*P* < 0.05). While only a 1.12-fold difference was detected between IR cells with and without insulin stimulation. The glucose consumption level differed significantly under insulin stimulation between control and IR cells (*P* < 0.05). The glucose uptake assay (Fig. 1C) showed similar results, accompanied by low levels of glucose uptake in IR cells. The expression level of GluT4 in the cell membranes of IR cells also significantly decreased compared with control cells based on immunofluorescence detection (Fig. 1D) and western blot (Fig. 1E,F) (*P* < 0.05). These results showed the successful establishment of an IR model using 3T3-L1 adipocytes.

The relative mRNA (Fig. 1G) and protein (Fig. 1H) levels of Twist 1 were confirmed in IR cells, which exhibited significant increases compared with control cells (*P* < 0.05). Application of Twist 1 shRNA induced a significant Twist 1 silencing effect, as Twist 1 decreased to (52.35 ± 6.46)% (Fig. 1I). Both glucose consumption (Fig. 1J) and glucose uptake tests (Fig. 1K) exhibited elevated glucose in the Twist 1-silenced cells compared with the NC cells (*P* < 0.05).

### Changes in mitochondria biosynthesis and function were involved in Twist 1-mediated IR of 3T3-L1 adipocytes

**Figure 2:**
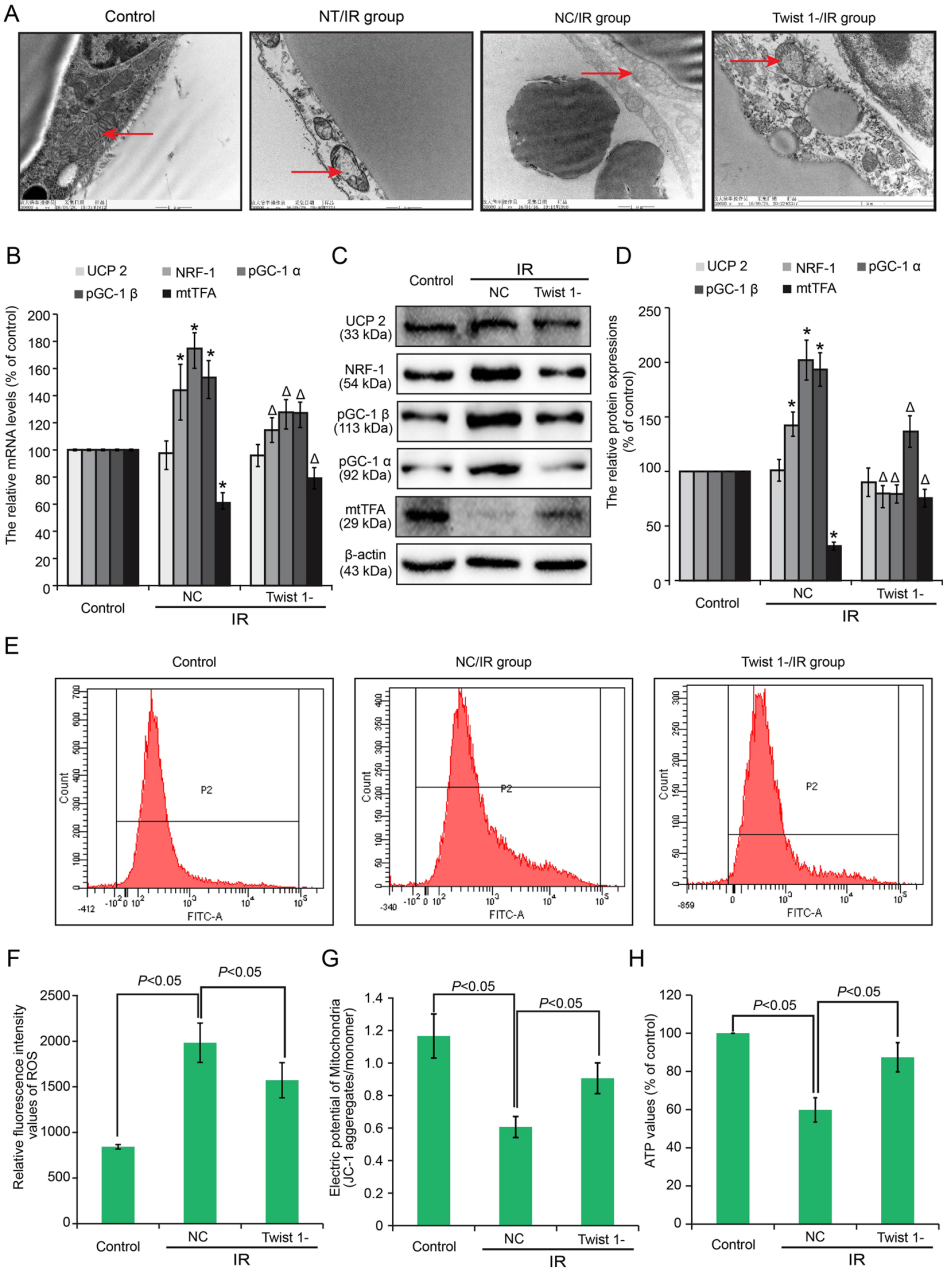
Changes in mitochondria biosynthesis and functions were involved in Twist 1-mediated IR of 3T3-L1 adipocytes. (**A**) Ultrastructural changes of mitochondria were analyzed via transmission electron microscopy in IR and Twist 1-silenced groups. NT/IR: IR group without further treatment (NT: No Treatment); NC/IR: IR group transfected with negative control. Twist 1-/IR: IR group transfected with a Twist 1-silencing virus. (**B**) The mRNA levels of UCP 2, NRF-1, pGC-1 α, pGC-1 β, and mtTFA in control, NC/IR and Twist 1-/IR 3T3-L1 cells. (**C**,**D**) Protein expression and semi-quantification of UCP 2, NRF-1, pGC-1 α, pGC-1 β, and mtTFA in control, IR and Twist 1-silenced cells. (**E**,**F**) ROS contents were assayed by flow cytometry, and the statistical analysis is shown. (**G**) MMP was assayed by flow cytometry. (**H**) ATP values were determined.

To further investigate the molecular mechanism of IR, we observed the ultrastructural changes in adipocytes based on TEM. Obvious ultrastructural changes were detected in high glucose/insulin stimulated cells, especially in the mitochondria, compared with control cells (Fig. 2A). The arrows indicate typical mitochondria morphology in control and IR cells. The mitochondria of IR cells exhibited marked damage, including mitochondrial swelling, altered integrity of the mitochondrial structure, and decreased mitochondrial matrix density. Many typical types of damage were observed particularly regarding changes in mitochondrial structure, such as reduced crest, disordered crest arrangement, or absence of the crest, etc. The mitochondria of Twist 1-silenced cells exhibited less damage compared with the NC/IR cells.

We next detected the changes in mitochondria biosynthesis-related genes (UCP 2, NRF-1, pGC-1 α, pGC-1 β, and mtTFA) and mitochondria function (ROS, MMP, and ATP). The mRNA and protein levels of mitochondria biosynthesis-related genes were determined based on real-time PCR (Fig. 2B) and western blot (Fig. 2C,D). Elevated NRF-1, pGC-1 α, and pGC-1 β and down-regulated mtTFA were confirmed in NC cells compared with control cells (^*^P < 0.05). These changes were markedly attenuated under Twist 1 silencing (^Δ^*P* < 0.05). However, no significant alteration in UCP2 expression was observed.

Furthermore, ROS content was assayed in 3T3-L1 adipocytes by flow cytometry. Figure 2E shows the ROS data obtained, and Fig. 2F shows the results of the statistical analysis of ROS content. The results revealing a marked increase in ROS levels in the NC/IR cells, which was attenuated by Twist 1 silencing (*P* < 0.05). High glucose/insulin stimulation decreased the MMP, and Twist 1 silencing restored the decrease in the MMP (*P* < 0.05) (Fig. 2G). Finally, ATP was decreased in NC/IR, and this decrease was also attenuated by Twist 1 silencing (Fig. 2H) (P < 0.05).

### The insulin signaling pathway and GluT4 expression were monitored in 3T3-L1 adipocytes

**Figure 3:**
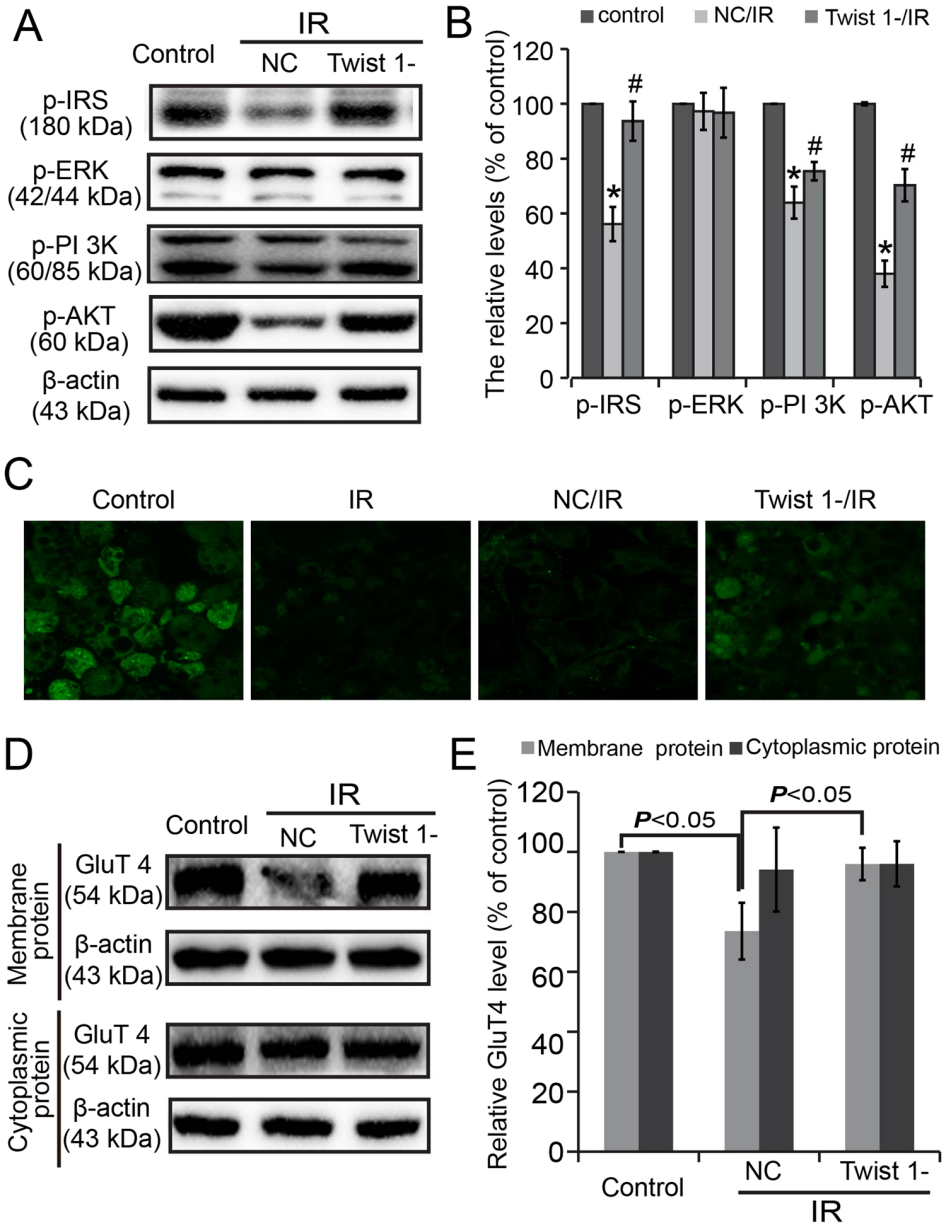
The insulin signaling pathway and GluT4 membrane translocation were monitored. (**A**) Insulin signaling pathway changes were assayed in cultured 3T3-L1 cells. (**B**) Semi-quantification of (**A**) based on Image J. (**C**) GluT4 membrane translocation was monitored by immunofluorescence. (**D**) Expressions of GluT4 in membrane and cytoplasmic proteins were assayed by western blot. (**E**) The semi-quantification of (**C**) using Image J.

For further clarification, the insulin signaling pathway involved in the IR state of the adipocytes was confirmed. Levels of p-IRS-1, p-AKT, and p-PI 3 K was decreased in NC/IR adipocytes compared with control cells (^*^P < 0.05), and Twist 1 silencing antagonized these changes (^#^*P* < 0.05). However, no obvious changes were found in p-ERK (Fig. 3A,B) (*P* > 0.05).

GluT4 membrane translocation was also monitored. Marked attenuation of the GluT4 membrane translocation inhibition induced by high glucose/insulin in Twist 1-silenced cells was assayed by immunofluorescence staining (Fig. 3C). The protein levels of GluT4 in the membranes significantly decreased in the NC/IR cells, and Twist 1 silencing attenuated this decrease (Fig. 3D,E) (*P* < 0.05).

### An IR model in C57/BL6J mice was induced by continuous HFD feeding for 16 weeks

We established an IR model in C57/BL6J mice by continuous HFD feeding for 16 weeks. The general information and plasma metabolic profile are shown in Table 1. The body weight, visceral and subcutaneous adipose weights of mice in HFD group all increased obviously compared with control (*P < 0.05). Insulin, glucose, TG, CHOL, HDL, and LDL were all significantly elevated in the HFD mice compared with control mice (*P < 0.05). NEFA obviously decreased in the HFD mice (*P < 0.05).

**Table 1:** General and metabolic parameters (mean ± STD).

Variables	control	HFD
weight (g)		
body weight (g)	30.85 ± 1.29	47.3 ± 0.85*
visceral adipose (g)	1.18 ± 0.17	6.75 ± 0.72*
subcutaneous adipose (g)	0.35 ± 0.04	2.88 ± 0.26*
insulin (pmol/L)	123.67 ± 16.60	239.31 ± 29.86*
Glu (mmol/L)	3.71 ± 0.44	11.15 ± 1.84*
TG (mmol/L)	0.63 ± 0.06	1.07 ± 0.42*
CHOL (mmol/L)	3.42 ± 0.36	6.17 ± 0.44*
HDL (mmol/L)	1.54 ± 0.12	2.13 ± 0.13*
LDL (mmol/L)	0.31 ± 0.03	0.755 ± 0.11*
NEFA (mmol/L)	1.09 ± 0.11	0.72 ± 0.09*

^*^VS control, *P* < 0.05.

**Figure 4:**
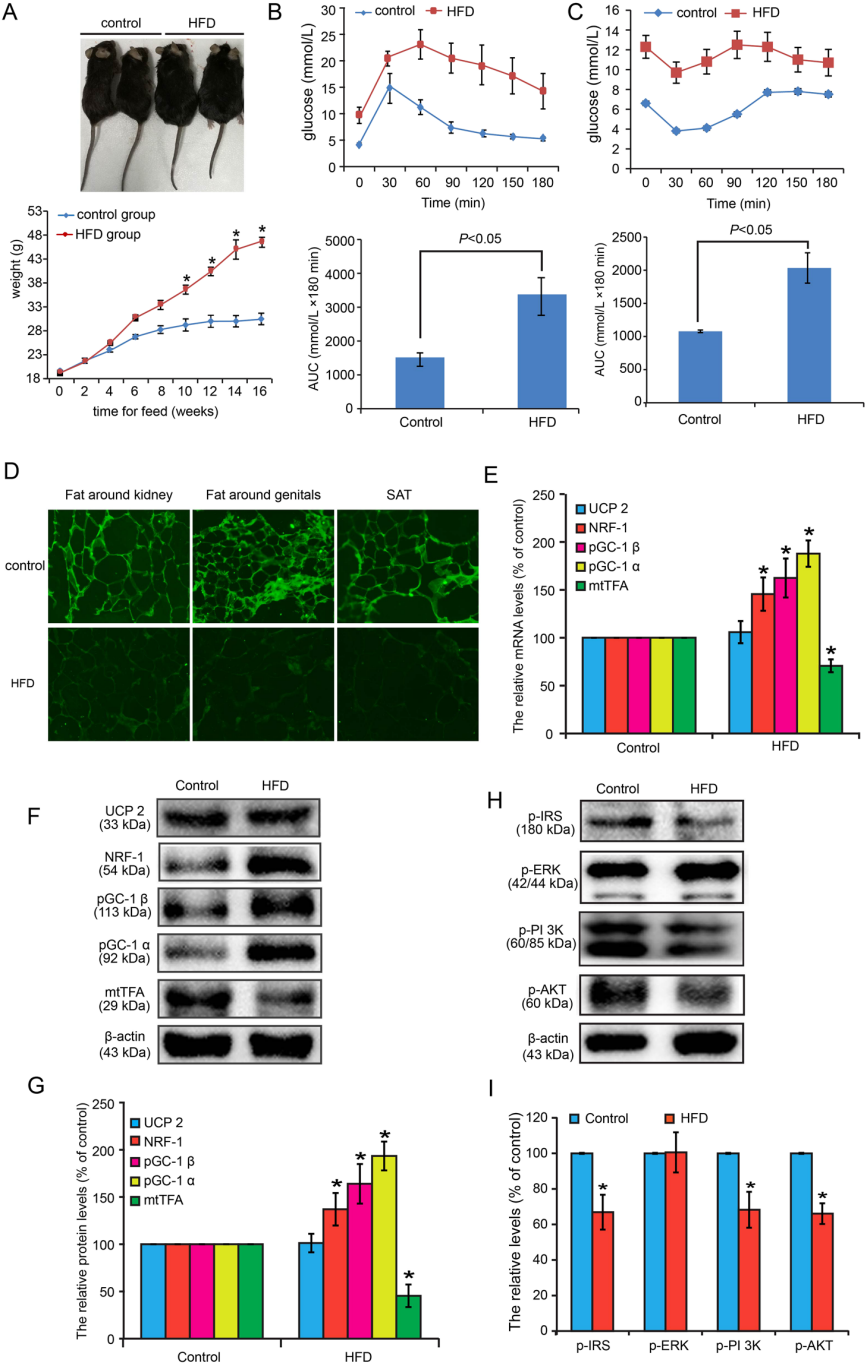
IR model in C57/BL6J mice was induced by continuous HFD feeding for 16 weeks. (**A**) Body shape and weight changes between control and IR groups. (**B**) IPGTT revealed that the sensitivity to glucose in IR was significantly decreased compared with controls. (**C**) IPITT revealed that the sensitivity to insulin in IR was significantly decreased compared with controls. (**D**) GluT4 membrane translocation in fat around the kidneys, fat around the genitals, and the SAT of IR mice were significantly decreased compared with control mice based on immunofluorescence by laser scanning confocal microscopy. (**E**) The mRNA levels of UCP2, NRF-1, pGC-1 α, pGC-1 β, and mtTFA were detected in mice perirenal adipose tissue. (**F**) The protein expressions of UCP 2, NRF-1, pGC-1 α, pGC-1 β, and mtTFA were monitored in mice perirenal adipose tissue. (**G**) The semi-quantification based on Image J software was conducted for (**F**). (**H**) Insulin signaling pathway in mice perirenal adipose tissue was also monitored after insulin (0.65 U/kg) stimulating for 10 min. (**I**) The semi-quantification based on Image J software was conducted for (**H**).

Figure 4A shows the body shape and body weight changes of the mice in each group. The mice in the IR group exhibited significant fat and weight increases (*P < 0.05). The IPGTT (Fig. 4B) revealed that the glucose in the mice with continuously fed was maintained at high levels not only during the fasting test but also under glucose stimulation. Significant differences were observed in the AUC between the IR group and the control group (*P* < 0.05). The IPITT (Fig. 4C) also showed that the glucose level in the IR mice was higher than that in in control mice, and the decreased glucose state under insulin (0.65 U/kg) stimulation required more time to recover according to the AUC (*P* < 0.05). Furthermore, GluT4 membrane translocation was monitored in fat around the kidneys, fat around the genitals, and SAT based on immunofluorescence by laser scanning confocal microscopy (Fig. 4D). In control mice, obvious green fluorescence was found in all AT samples, while the fluorescence intensity of the IR mice markedly was decreased.

We then detected the protein and mRNA levels of mitochondria biosynthesis-related genes, including UCP 2, NRF-1, pGC-1 α, pGC-1 β, and mtTFA in mice perirenal adipose tissue. As shown in Fig. 4E, the mRNA levels of NRF-1, pGC-1 α and pGC-1 β were all up-regulated in perirenal adipose tissue of HFD-feeding mice, and mtTFA decreased (*P < 0.05). No significant change was observed for UCP2 transcription. The protein expressions of UCP 2, NRF-1, pGC-1 α, pGC-1 β, and mtTFA in mice perirenal adipose tissue showed similar results with mRNA, with significant increase of NRF-1, pGC-1 α and pGC-1 β, decease of mtTFA (*P < 0.05) (Fig. 4F,G).

Insulin signaling pathway in mice perirenal adipose tissue was also monitored after insulin (0.65U/kg) stimulating for 10 min. Levels of p-IRS-1, p-AKT, and p-PI 3 K was decreased in HFD-feeding mice compared with control (^*^P < 0.05). However, no obvious changes were found in p-ERK (Fig. 4H,I) (*P* > 0.05).

### Twist 1 silencing attenuated the IR state of C57/BL6J mice, and Wort. application blocked this attenuation effect

**Figure 5:**
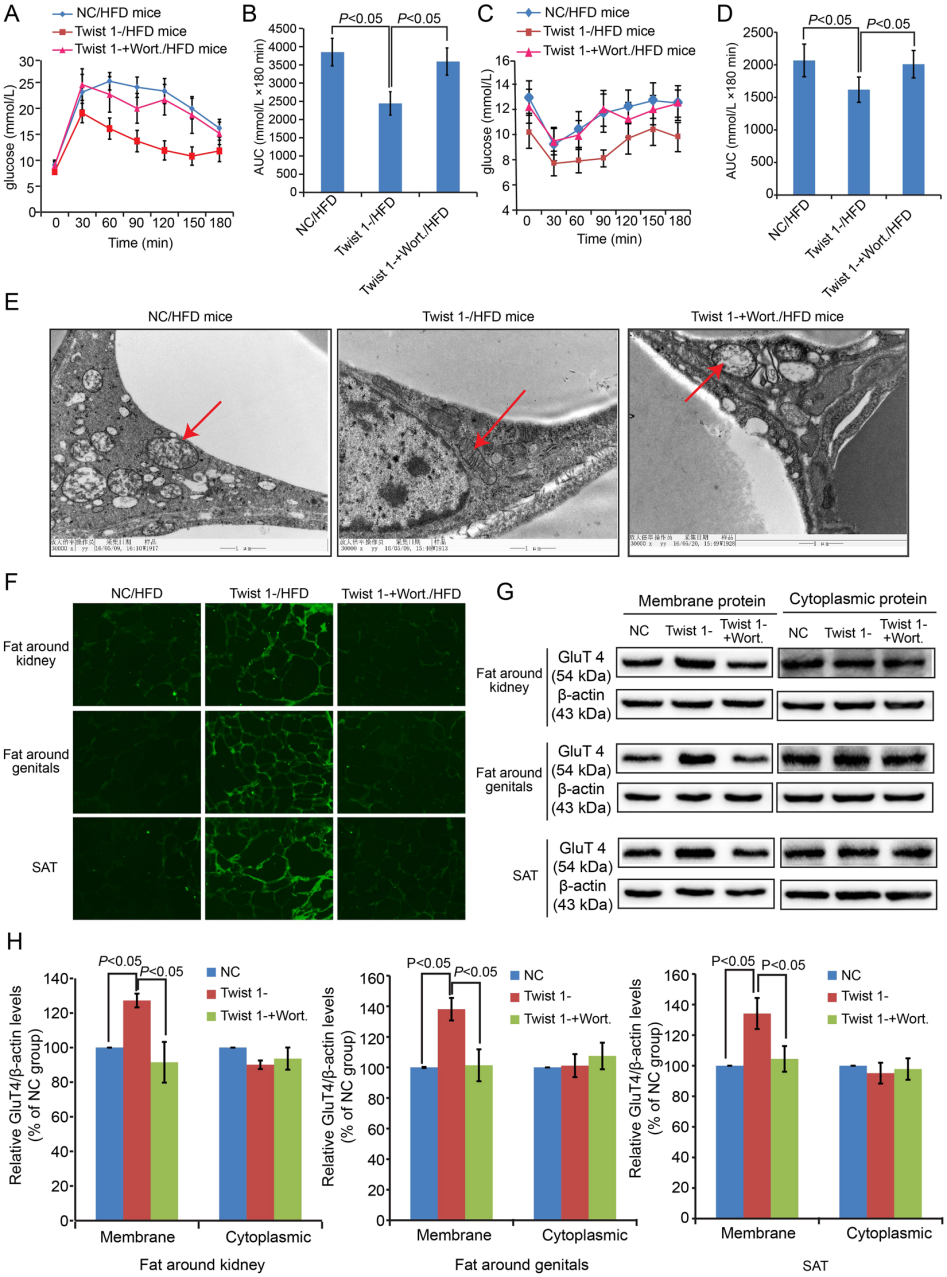
Twist 1 silencing attenuated the IR state of C57/BL6J mice, and application of the IRS/PI3K/AKT/GluT4 pathway inhibitor Wort. blocked this attenuation effect. (**A**,**B**) IPGTT revealed the attenuation of IR induced by HFD by Twist 1, and application of Wort. blocked this attenuation effect. (**C**,**D**) IPITT test also revealed the attenuation of IR induced by HFD by Twist 1, and application of Wort. blocked this attenuation effect. (**E**) Ultrastructural changes of mitochondria showed the protective function of Twist 1 silencing, and Wort. blocked this protection. (**F**) Twist 1 silencing enhanced GluT4 membrane translocation in fat around the kidneys, fat around the genitals, and SAT inhibited by HFD, which was blocked by Wort. based on immunofluorescence. (**G**) Western blot further proved the changes in GluT4 in membrane protein in fat around the kidneys, fat around the genitals, and SAT. (**H**) The semi-quantification based on Image J software was conducted for the relative expressions of GluT4 in both membrane and cytoplasmic proteins.

To confirm the attenuation effect of Twist 1 silencing in IR, we introduced Wort., an inhibitor of the PI 3 K/AKT pathway, via a Twist 1-silencing virus. Figure 5A,B shows the IPGTT results. The glucose levels under Twist 1 silencing decreased significantly compared with the NC mice, which was blocked by Wort. application. The IPITT (Fig. 5C,D) also proved the attenuation of the IR state by Twist 1 silencing and verified that Wort. application blocked this effect. TEM was also conducted in the perirenal AT of C57/BL6J mice (Fig. 5E). The arrows indicate typical mitochondria morphology in each group. Obvious mitochondrial damage was observed in the NC/HFD mice, characterized by swollen mitochondria, decreased mitochondrial matrix density, and disordered, reduced or absent crest, etc. Mitochondrial damage in the Twist 1-silenced group was eased regarding both mitochondrial morphology and structure. In the Wort. application group, the mitochondrial damage was not obviously changed compared with the NC mice. Furthermore, GluT4 membrane translocation was monitored in the fat around the kidneys and genitals and via SAT based on immunofluorescence by laser scanning confocal microscopy (Fig. 5F) and western blot (Fig. 5G). The semi-quantification based on Image J software showed that the expression of GluT4 in the membrane in the NC group and in the Twist 1-silenced plus Wort group exhibited similar low levels compared with the Twist 1-silenced group (Fig. 5H), with statistical significance (*P* < 0.05).

## Discussion

In the present study, we provide evidence that Twist 1 silencing can relieve the IR phenotype of both 3T3-L1 adipocytes induced by high glucose/insulin stimulation and IR model C57/BL6J mice induced by HFD. Our results suggest that mitochondrial dysfunction was involved in Twist 1-mediated IR based on detections of mitochondrial ultrastructure, mitochondrial synthesis-related gene expression (UCP 2, NRF-1, pGC-1 α, pGC-1 β, and mtTFA), ROS generation, MMP alteration, and ATP content. Furthermore, we propose that the IRS/PI3K/AKT/GluT4 pathway is involved in the downstream of insulin signaling transduction in Twist 1-mediated IR. In summary, the present results suggest that Twist 1 silencing is protective against IR in adipocytes by relieving mitochondrial dysfunction and further that the IRS/PI3K/AKT/GluT4 insulin signaling pathway is involved.

Twist 1 has been widely studied for its function as a potential regulator of AT, including white adipose tissue (WAT) and brown adipose tissue (BAT)^28,29^. In the present study, we focused on the role of Twist 1 in IR, which is highly important in AT. In fact, Twist 1 was previously reported as early as in 2009 to regulate the metabolism of WAT and BAT^12^. Twist 1 has been suggested as a negative feedback regulator that finely tunes PGC1α/PPARδ-controlled brown fat metabolism to ensure energy homeostasis^30^. Additionally, reduced Twist 1 silencing to fatty acid oxidation has also been reported^13,31,32^. These studies implicated the potential role of Twist 1 in AT. In the present study, we focused on the role of Twist 1 in IR of 3T3-L1 adipocytes induced by high glucose/insulin stimulation and of IR model C57/BL6J mice induced by HFD.

We next questioned whether the role of Twist 1 in IR is protective or destructive. Our results suggest that Twist 1 silencing is protective against IR in both 3T3-L1 adipocytes and in C57/BL6J mice. In 2010, Pettersson *et al*. noted that gene silencing of Twist 1 in human white adipocytes had no effect on glucose transport, although no graphs were shown^31^. The same research team later reported that low levels of Twist 1 were associated with decreased insulin sensitivity *in vivo* (measured as HOMA-IR)^33^. Both reports were inconsistent with our results, which may be due to the following differences:

1. Different types of samples: the samples used in Pettersson’s study were obese humans (BMI > 30) and cultured human differentiated adipocytes, while the samples in the present study were cultured mouse 3T3-L1 adipocytes and IR model C57/BL6J mice induced by HFD feeding. This may be one main reason for the inconsistencies. Similar inconsistencies were observed in other studies because of the use of different types of samples^31^.
2. Different methods used to induce the IR phenotype: The method used to establish the IR model differed, and high glucose/insulin stimulation and HFD feeding were the methods used to induce IR in the present study. Although an IR phenotype can be induced in multiple ways, the accompanying microenvironment differs for the various ways to induce IR. Even for the similar HFD-induced IR model, different calories level, different HFD-feeding time, or different animal species could all bring different microenvironment of the body, which were all closely related with the results. Therefore, we cannot completely rule out inconsistences due to different microenvironments. Furthermore, we maintain the view that, even for the same disease model, results may be incomparable because of the different methods used to develop the model.
3. A negative correlation between Twist 1 mRNA and HOMA-IR values does not indicate a destructive role of Twist 1 in IR. This observed relationship does not directly support a causative relationship between Twist 1 and IR, although it strongly suggests a contribution of Twist 1 to the pathogenesis of IR.

Mitochondrial dysfunction has been implicated in the development of diabetes and IR^34,35^. In the present study, we considered this point in further determining the mechanism of Twist 1-associated IR. We aimed to clarify whether mitochondrial dysfunction was involved in Twist 1-associated IR. Obvious mitochondrial ultra-structural damage was observed in IR adipocytes. Additionally, our *in vitro* results support our TEM results, with mitochondria biosynthesis inhibition, elevated ROS, and decreased MMP and ATP in IR cells. Twist 1 silencing induced blocked these changes. In fact, the relationship between mitochondrial dysfunction and IR remains controversial, especially for the causative relationship between mitochondrial dysfunction and IR. Mitochondrial dysfunction was first described for glucose intolerance in skeletal muscle^36^, and the debate regarding mitochondrial dysfunction and IR is ongoing^37^. In adipocytes, mitochondrial dysfunction sometimes induces IR. The application of Celastrol, potent NF-κB inhibitor, was reported to modulate mitochondrial dysfunction-induced IR in 3T3-L1 adipocytes^38^. Mitochondrial dysfunction was also believed to constitute a mechanistic link between various genes, such as Nat1, and IR^39^. Our results complemented the study of mitochondria in IR and support a causative role of mitochondria dysfunction in IR occurrence.

Additionally, it is worth adding that the definition of “mitochondria dysfunction” is very broad, including changes in mRNA and protein levels of mitochondrial markers^40,41^, alterations of the enzymatic activity of key components of mitochondria-driven oxidation^42^, mitochondrial size and shape changes^43^, and substrate oxidation^44^, etc. The differences in selected indicators and detection methods can lead to varying results. In the present study, we refer to “mitochondria dysfunction” as mitochondrial damage in shape, abnormal expression of mitochondria biosynthesis-related genes (UCP 2, NRF-1, pGC-1 α, pGC-1 β, and mtTFA), elevated ROS, and decreased MMP and ATP.

The insulin signaling pathway is a core and key link inducing glucose transport in IR. Generally, insulin first binds with its receptor located in the membrane of adipocytes, which activates the β subunit of the insulin receptor. Activation of the β subunit phosphorylates the insulin receptor substrate 1 (IRS-1), which can further activate or bind with proteins containing an SH2 domain, such as phosphatidylinositol 3-kinase (PI3K) and Grb2. PI (3, 4) P_2_ and PI (3, 4) P_3_ are then formed under the catalysis of PI3K, both of which are the anchorages of intracellular signaling proteins, and activates them. The main downstream signaling pathways are (1) activation of Bruton’s tyrosine kinase (BTK) and phospholipase Cγ (PLCγ), inducing the phosphatidylinositol pathway and (2) activation of the phosphoinositol-dependent kinase (PKD1) and then activation of the PKB (also termed Akt), which mediates the membrane transport of GluT4^45–48^. We detected changes in the insulin signaling pathway, including p-IRS-1, p-ERK, p-AKT, and p-PI3K. The results suggest that the IRS/PI3K/AKT/GluT4 pathway, but not p-ERK, is involved in Twist 1-mediated IR.

It is worth mentioning that the harmful effect accompanied with HFD feeding can’t be ignored, which may also be responsible for some inconsistence of different research. As we know, HFD was reported widely in research for obesity, diabetes, and metabolic disease. This means that continuous feeding of HFD could bring various changes to the body. The harmful effect of HFD is based on the overload of palmitic acid, and brings lipotoxicity, glucotoxicity, oxidative stress, and inflammation to the body^49,50^. HFD was reported significantly increased hepatic total fat, enhanced oxidative stress, and reduced the desaturation capacity of the liver in mice subjected to high fat diet in 2015^51^. A lipid profiles disorder was also found under HFD induction, and further disturbances in kidney and liver function were showed recently^52^. HFD-induced lipid accumulation, cardiac dysfunction, pancreas lipotoxicity and brain lipotoxicity all could be found^53–56^. Also, oxidative stress status mainly characterized by increased lipoperoxidation was believed responsible for nearly all of the tissue lipotoxicities^51,57^. Fortunately, the liver and renal lipotoxicity induced by HFD is exactly one of our studies undergoing now, and further data will be shown in our next work.

**Figure 6:**
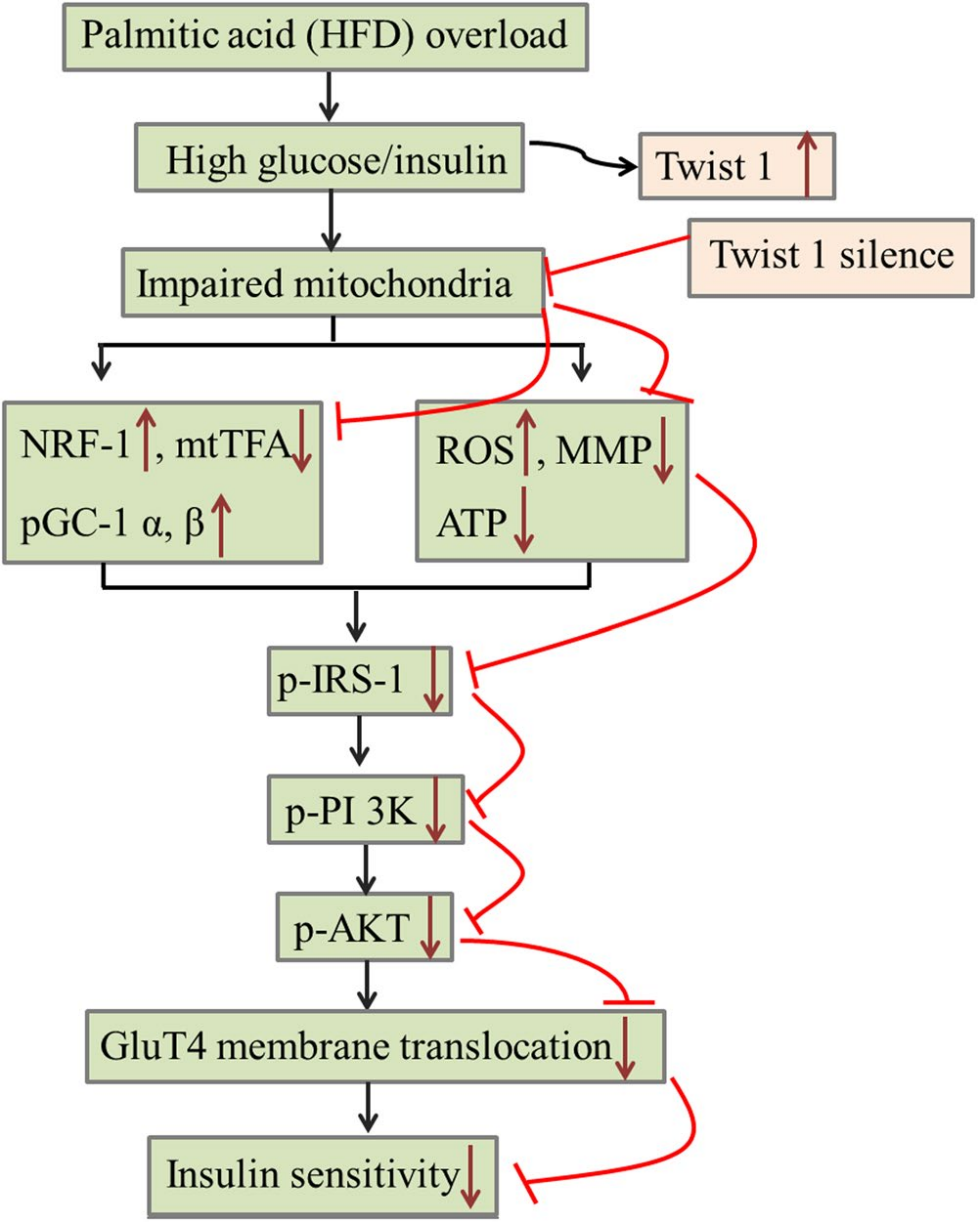
A model for the role of Twist 1 in IR and a possible molecular mechanism.

In summary, the present study provides evidence of the protective role of Twist 1 silencing in relieving IR of 3T3-L1 adipocytes induced by high glucose/insulin stimulation and of IR model C57/BL6J mice induced by HFD feeding. The molecular mechanism is related with mitochondrial dysfunction and abnormal insulin signaling transduction, mainly in the p-IRS/p-PI3K/p-AKT/GluT4 pathway (Fig. 6). The present results suggest that Twist 1 silencing is a protective strategy against IR of adipose and provide important insight into the related molecular mechanism. Further studies must be performed to reveal more details regarding the regulation involved in Twist 1 and adipocyte IR, and upcoming studies will provide further valuable information for Twist 1 function and IR regulation.

## Methods

### Cell lines and mice

C57BL6J mice (male, six weeks) were purchased from the Experimental Animal Center of Shandong University. 3T3-L1 mouse embryo fibroblasts were obtained from ATCC (Rockefeller, Maryland, USA).

### Reagents

A HFD diet (60 kcal %, D12492) and a basal diet (10 kcal %, D12450B) were acquired from Research Diets. Bovine serum, fetal bovine serum (FBS), and Dulbecco’s modified Eagle’s medium (DMEM) were all purchased from GIBCO (Invitrogen, CA, USA). Insulin, 3-isobutyl-1-metyl-xanthine (IBMX), dexamethasone (DXM), Oil red O working solution, Wortmannin (Wort.) and the primary antibodies for anti-Twist 1 and GluT4 were Sigma products (Merck, German). Primary antibodies for anti-UCP 2, NRF-1, pGC-1 α, pGC-1 β, β-actin, and mtTFA, HRP-conjugated secondary antibodies, and IgG-FITC were all obtained from Abcam. Primary antibodies for anti-p-IRS-1, p-ERK, p-AKT, and p-PI 3 K were CST products. 2-deoxy-D-[1,2-,3 H]-glucose was obtained from Perkin Elmer. The lentiviral vectors pGLV3/H1/GFP + Puro (LV3) and recombinant vectors LV3/Twist 1 shRNA were acquired from Gene Pharma (Shanghai, China). NE-PER^TM^ Nuclear and Cytoplasmic Extraction Kits, Mem-PER^TM^ Plus Membrane Protein Kits, and Mitochondria Isolation Kits were all kindly supplied by Pierce (Thermo Scientific). SYBR® Green Real-time PCR Master Mix (QPK-201 T) was purchased from TOYOBO. All other reagents were obtained from Sigma.

### *In vitro* experiments based on cultured 3T3-L1 adipocytes

#### Differentiation of 3T3-L1 preadipocytes and Oil red O staining

3T3-L1 preadipocytes were cultured and differentiated as described previously based on insulin, IBMX and DXM exposure^58,59^. Mature adipocytes were treated for experiments during the day 12 of differentiation when the cells exhibited biochemical and morphological properties. Mature 3T3-L1 adipocytes were stained with Oil red O based on common procedures.

#### Establishment of the IR model based on 3T3-L1 adipocytes

3T3-L1 adipocytes were first adjusted to an environment of low-glucose DMEM (1 g/L) with 10% FBS for 2 days and then were cultured in low-glucose serum-free DMEM with 0.2% bovine serum albumin (BSA) overnight. Insulin resistance models were established by stimulating mature adipocytes with high-glucose DMEM containing 10% FBS and 10^−7^ M insulin for 2 days. Cells as a negative control (NC) were maintained in low-glucose DMEM supplemented with 10% FBS and without insulin. The media were changed every day.

#### Twist 1 shRNA transfection

NC/LV3 or LV3/Twist 1 shRNA was used to transfect the mature adipocytes before low sugar adaptation. Generally, the proportion of shRNA and high-glucose 10% FBS-DMEM was 1:20. Following 8–12 h of incubation, the transfected medium was replaced with updated 10% FBS-DMEM, and the cells incubated for 2 days.

#### Glucose consumption test

Adipocytes were stimulated with low-glucose 10% FBS-DMEM with 10^−7^ M insulin for 4 h, whereas there was no insulin in the media of the NC group compared to the stimulated groups. Glucose concentrations in the medium and in each group of cells were determined simultaneously. The difference in glucose between the medium and the cells represented the glucose consumption level.

#### Glucose uptake assay

3T3-L1 cells were cultivated with or without 100 nM insulin in Krebs-Ringer phosphate buffer (KRPB) buffer for 30 min after being washed three times gently with KRPB buffer (140 mM NaCl, 5 mM KCl, 1 mM CaCl_2_, 2.5 mM MgSO_4_, 5 mM NaHCO_3_, PH 7.4). KRPB buffer plus 0.5 μCi/ml 2-deoxy-[^3^H]-D-glucose and 0.1 mM 2-deoxy-d-glucose as atopic glucose uptake was added and incubated for 10 min at 37 °C with KRPB buffer containing 10 μM cytochalasin D as a non-atopic control. Glucose uptake was ended up by washing quickly three times with precooled PBS containing 10 mM glucose. Cells lysates were harvested with 0.5 mL 0.1 M NaOH per well for 2 h. Before assaying in a liquid scintillation counter, cells lysates were mixed with 5 mL scintillation fluid. The final amount of glucose uptake differed between the atopic and non-atopic groups.

#### Flow cytometer analysis

Reactive oxygen species (ROS) and the mitochondrial membrane potential (MMP) were detected using specific assay kits from Beyotime (Shanghai, China). Generally, cells were collected by trypsin digestion and washed with PBS. Then, the cells were divided into three groups: a negative control group to which an equal amount of PBS instead of the staining dye was added, a positive control group to which an equal amount of Rosup (50 mg/mL) for ROS or carbonyl cyanide m-chlorophenylhydrazone (CCCP, 10 mM) for MMP was added, and a sample group to which 1 μL fluorescence probe DCFH-DA for ROS or CCCP (10 mM) for MMP detection was added. The tubs were incubated for 20 min at 37 °C and intermittently mixed 3–4 times. After washing twice with serum-free DMEM, cells were suspended in suitable PBS for flow cytometry analysis.

#### Adenosine 5′-triphosphate (ATP) assay

ATP levels were determined using an ATP Assay Kit from Beyotime (Shanghai, China). Briefly, the culture medium was removed, and cells were exposure to 200  μL lysis buffer for full lysis by vortex gently. The cells were centrifuged at 4 °C for 5 min (12000 g), and the supernatant was collected for further detection. Then, 100 μL ATP working solution was added to each well, and cells were maintained at room temperature for 3–5 min to deplete the background ATP. Then, 10 μL sample or standard solution was added to each well and quickly mixed. The CPM value was assayed under a liquid scintillation counter. The protein concentration was quantified using a BCA Protein Assay Kit. The levels of ATP were finally calculated as nmol/mg protein.

## Animal Studies

### Group design and treatment

Seventy-five adult male C57/BL6 mice were first fed for one week of adaptation by basal diets and were divided into five groups (15/group): (1) a control group fed the basal diet; (2) a HFD group fed the HFD; (3) a Twist 1-silenced group (Twist 1-/HFD), fed the HFD and injected with LV3/Twist 1 shRNA lentivirus in the first week of induction (tail intravenous injection, t.iv.); (4) a NC/LV3 group (NC/HFD) fed the HFD and injected with LV3 blank lentivirus in the first week of induction (t.iv.); and (5) a Twist 1-silenced with Wort. treatment group (Twist 1-/Wort./HFD) fed a HFD and injected with LV3/Twist 1 shRNA lentivirus (t.iv.) and Wort. (3 mg/kg, intraperitoneal injection, i.p) in the first week of induction. Animals were free for water and diet during the feeding period. The weight of each animal was determined once per week for the duration of the experiment.

### Collection and detection of C57/BL6 mice samples

Blood and fat tissues were collected after deep anesthesia with 0.3% pentobarbital sodium (1 ml/kg, i.p). Serum was obtained by centrifugation (500 × g, 4 °C, 10 min), and cholesterol (CHOL), triglyceride (TG), glucose (GLU), high-density cholesterol (HDL), low-density cholesterol (LDL), and non-esterified fatty acid (NEFA) contents were measured using a Beckman DXC 800 analyzer (Beckman Coulter, Inc., California, USA). Insulin levels were detected using an ELISA kit. Subcutaneous adipose tissue (AT) samples from around the genitals and around the kidney were collected and quickly frozen by immediate immersion in liquid nitrogen. They were then stored at −80 °C prior to protein extraction. The animal experiments were performed in accordance with the ‘Principles of Laboratory Animal Care’ established by the National Institutes of Health and were approved by the Animal Care and Use Committee of Shandong University.

### Intraperitoneal glucose tolerance test (IPGTT) and intraperitoneal insulin tolerance test (IPITT)

After fasting overnight for IPGTT or six hours for IPITT, the mice were injected with 50% glucose (2.0 g/kg, i.p) or insulin (0.65 U/kg, i.p). Glucose levels were measured in blood collected from the tail vein at 30, 60, 90, 120, 150, and 180 min using a blood glucose meter (Bayer, Germany). The area under the curve (AUC) was compared among different groups to compare IPGTT and IPITT.

### Rapid frozen sections of fat tissue

Fat tissue was rapidly immersed in liquid nitrogen after removing from the mice. For frozen sections, the temperature of the workspace of the rapid frozen slicing machine was maintained at −40 °C and the slice thickness was 7–8 μm. Successful slices were placed in 4 °C for 1–2 h and then moved to −80 °C for storage.

### Transmission electron microscopy (TEM) analysis

We collected 3T3-L1 adipocytes from control cells, IR cells with no further treatment (NT/IR), IR cells infected with the NC virus (NC/IR), and IR cells infected with the Twist 1 shRNA virus (Twist 1-/IR). Additionally, perirenal AT from NC/HFD, Twist 1-/HFD and Twist 1-/Wort./HFD mice fed the HFD for 16 weeks were collected for TEM analysis of the ultrastructural changes of adipocytes. Generally, the cells or perirenal AT were trimmed to the appropriate size using a sharp blade and immersed quickly into cooled glutaraldehyde for fixation. Following embedding, ultra-thin slices were prepared, and TEM was conducted by JiNan WeiYa Bio-technology Co., Ltd. (Jinan, Shandong, China), which specializes in biomedical ultrastructure and methodology.

### Immunofluorescence staining

Immunofluorescence staining was conducted based on standard procedures for the GluT4 membrane translocation analysis^60^. Briefly, cells or fat tissues were blocked with 10% donkey serum for 30 min at room temperature without membrane rupture treatment by Triton. The primary antibody GluT4 (1:1000) was used to determine membrane distribution. Equal PBS was added instead of GluT4 as a negative control. The cells or fat tissues were observed using a laser scanning confocal microscope.

### Real-time PCR detection

RNA extraction and real-time PCR were conducted and quantified as described previously^61^. All experiments were conducted at least three times. Data were normalized to GAPDH expression. The primers used here were designed by Primer 5.0 Software, and the sequences were as follows:

Twist 1: Forward: 5′-GGACAAGCTGAGCAAGATTCA-3′

Reverse: 5′-CGGAGAAGGCGTAGCTGAG-3′

UCP 2: Forward: 5′-CAGCGCCAGATGAGCTTTG-3′

Reverse: 5′-GGAAGCGGACCTTTACCACA-3′

PGC-1α: Forward: 5′-AAGTGGTGTAGCGACCAATCG-3′

Reverse: 5′-AATGAGGGCAATCCGTCTTCA-3′

PGC-1β: Forward: 5′-TGACGTGGACGAGCTTTCAC-3′

Reverse: 5′-GGGTCTTCTTATCCTGGGTGC-3′

mtTFA: Forward: 5′-ATTCCGAAGTGTTTTTCCAGCA-3′

Reverse: 5′-TCTGAAAGTTTTGCATCTGGGT-3′

NRF-1: Forward: 5′-CGGAAACGGCCTCATGTGT-3′

Reverse: 5′-CGCGTCGTGTACTCATCCAA-3′

GAPDH: Forward: 5′-TGGCCTTCCGTGTTCCTAC-3′

Reverse: 5′-GAGTTGCTGTTGAAGTCGCA-3′

### Extractions of nucleoprotein, membrane protein, and mitochondrial protein

Adipocytes were generally collected by washing twice with ice-cold PBS and scrapping into the appropriate lysis buffer. Each protein was extracted according to the instructions of the respective reagent kit.

Briefly, for nucleoprotein extraction, cells were washed three times, harvested with PBS and centrifuged at 500 × g for 5 min in 1.5-ml microcentrifuge tubes. Next, ice-cold CERI was added to the cells, which were as dry as possible. The cells were vortexed vigorously on the highest setting for 15 s and then incubated on ice for 10 min. Then, ice-cold CERII was added, and the cells were vortexed for 5 s and incubated for 1 min. The tube was centrifuged at 16,000 × g for 5 min, and the supernatant was transferred to obtain the cytoplasmic protein. Finally, ice-cold NER was added, and the tube was vortexed vigorously on the highest setting for 15 s every 10 min for a total of 40 min on ice, centrifuged at 16,000 × g for 5 min. The supernatant was transferred to obtain the nuclear protein.

For membrane protein extraction, permeabilization buffer was added to the cells, briefly vortexed, incubated for 10 min at 4 °C under constant mixing, and centrifuged at 16,000 × g for 15 min. The supernatant containing the cytoplasmic protein was transferred, and then solubilization buffer added, vortexed briefly, incubated for 30 min at 4°3 under constant mixing and centrifuged at 16,000 × g for 15 min to obtain the supernatant containing the membrane-associated protein.

For mitochondrial protein extraction, a mitochondria isolation kit was used first to isolate mitochondria. Manual homogenization was critical to yield the maximum mitochondria with minimal damage. Then, lysis of isolated mitochondria by RIPA was carried out to yield mitochondrial protein.

### Western blot analysis

Nucleoprotein was used for Twist 1, pGC-1 α, pGC-1 β, and NRF-1 assays. Membrane-associated protein was used for GluT4 membrane translocation detection, and mitochondrial protein was used to determine the biosynthesis-related genes UCP 2 and mtTFA. Phosphorylation of insulin signaling pathway-related proteins was detected in cytoplasmic proteins supplemented with complex phosphatase inhibitors. Protein concentration quantification and western blot analysis were conducted via typical procedures^62^. The suitable primary antibody concentrations for Twist 1 (26 kDa), GluT4 (54 kDa), UCP 2 (33 kDa), NRF-1 (54 kDa), pGC-1 α (92 kDa), pGC-1 β (113 kDa), mtTFA (29 kDa), p-IRS-1 (180 kDa), p-ERK (42/44 kDa), p-AKT (60 kDa), and p-PI 3 K (60/85 kDa) were all 1:1000. The immunoreactive bands were visualized using an enhanced chemiluminescence kit in a chemiluminescence system.

### Statistical analyses

ImageJ software was used to quantify the western blot bands. The relative gray values of each band were calculated based on the results of three replicates. ROS, MMP, and ATP measurements were repeated more than three times. All data are shown as the mean ± SD, and the data were analyzed using SPSS 17.0. T tests were used for comparisons between two groups. One-way analysis of variance (ANOVA) was performed to compare more than two groups, and Dixon’s *q* tests were used for further pairwise comparisons. *P* < 0.05 was considered significant.
